# Mining of Candidate Genes Associated with Leaf Shape Traits in Grapes

**DOI:** 10.3390/ijms252212101

**Published:** 2024-11-11

**Authors:** Chuan Zhang, Vivek Yadav, Liwen Cui

**Affiliations:** The State Key Laboratory of Genetic Improvement and Germplasm Innovation of Crop Resistance in Arid Desert Regions, Key Laboratory of Genome Research and Genetic Improvement of Xinjiang Characteristic Fruits and Vegetables, Institute of Horticultural Crops, Xinjiang Academy of Agricultural Sciences, Urumqi 830091, China; 2016204005@njau.edu.cn

**Keywords:** grape, leaf shape, Tomato Analyzer, genome-wide association study, candidate gene

## Abstract

As the most important organ for photosynthesis, leaves provide the main energy source for plant growth. Leaf traits affect light energy utilization and, thus, plant development and biomass. Given the high morphological variability of leaves between and within grape genotypes, phenotypic analysis is challenging. This study first evaluated leaf shape trait parameters using a specific leaf profile and area analyzer, along with genome-wide association study (GWAS) analyses, to identify additional candidate genes related to grape leaf shape traits. In the two-year analysis, 89 single-nucleotide polymorphisms (SNPs) were found to be significantly associated with leaf shape traits. These SNP loci were distributed on 18 chromosomes, of which chromosome 15 had the most relevant SNPs. We found that leaf shape-associated genes included mainly plant hormone-, ubiquitin ligase-, serine/threonine protein kinase-, transcription factor-, and cell wall metabolism-related genes. By analyzing the expression of these candidate genes on the chip, we found that they exhibited diverse expression levels in leaves at different developmental stages (young, mature, and senescent). This suggests that these genes could be considered candidates for grape leaf improvement.

## 1. Introduction

Plants have diverse leaf shapes, with variations both among and within different species, developmental stages, and growth conditions [1,2]. Leaves can be classified as either simple or complex based on their morphology [3]. In most plant species, the leaf is a flattened, typically green, blade-like structure attached directly to a stem or via a stalk [4]. Light energy capture occurs on the upper leaf surface, while gas exchange and transpiration are confined mostly to the underside of leaves [4].

As the primary organ for photosynthesis, leaves are not only the main energy source for plant growth but also play a crucial role in supporting human nutrition by forming the base of the food chain [5,6]. Leaf dimensions influence the efficiency of light energy utilization, which affects plant growth and biomass production. Additionally, leaves play a crucial role in physiological processes such as photorespiration, transpiration, and temperature regulation in plants [7]. As a result, leaf dimensions also play a role in a plant’s adaptability and its response to stress.

Grape (*Vitis* spp.) leaves have five main veins arranged in a palmate pattern [8]. Despite this consistency of grape leaf architecture, morphology is diverse [8]. Leaves vary widely, ranging from simple forms to highly divided or compound structures, with a variety of shapes and degrees of lobing in between. The angle and lengths between the upper (distal) and lower (proximal) side veins contribute to this diversity, resulting in leaf shapes such as round (orbicular), kidney-shaped (reniform), and heart-shaped (cordate).

The leaves of the genus *Vitis* show an astonishing range of variation in leaf shape, which makes the genus well suited for exploring potential shapes created during evolution and development [9]. Researchers specializing in the genus *Vitis* have examined variations in dimensions to classify nearly 60 distinct species [10,11].

The economic motives and the desire to capture the qualities of a location’s terroir drive researchers and farmers to select and plant genotypes that are best adapted to specific environments. This results in better yields, higher-quality produce, and more efficient farming tailored to the land’s unique characteristics. A key example highlighting the value of ampelography emerged during France’s phylloxera crisis in the late 19th century [12]. The leaf morphology of more than 1200 grape varieties was comprehensively graphically assessed, and the genetic basis of leaf shape was demonstrated [13]. Elliptical Fourier descriptors and Procrustes analysis of various leaf traits, including primary vein branching points, sinuses, and leaf-tip specifications, offer a thorough approach to analyzing leaf shape. Collectively, these studies establish a solid genetic foundation for leaf shape and enable the quantitative measurement of natural variations in grape leaf morphology. Targeting grape leaves for breeding could be a promising strategy to help vineyards adapt to the anticipated impacts of climate change [13].

An important challenge in agriculture sciences, sustainable agriculture, phytomedicine, and biodiversity conservation is the ability to quickly identify specific species among the 260,000 cultivated species [14]. As an important topic in the field of agricultural information, plant species classification has attracted considerable attention from digital imaging experts in recent years [15]. The purpose of plant species classification is to assign test sample plants to a species based on morphological characteristics, including roots, stems, leaves, flowers, and fruit [16]. Genetic resources and plant breeding communities still rely on traditional descriptors to characterize the agronomic performance of horticultural crops [17]; however, traditional phenotypes limit the study of the detailed characteristics of plant morphology. The swift advancement and widespread adoption of machine-based technology are transforming the field of plant science. Advanced imaging tools and machine learning models enable broad access to plant information, support efficient data management, and promote collaboration between botanists and machine learning researchers, enhancing the analysis and discovery of distinctive plant features [2]. Leaves have valuable discriminant information that can be used for plant classification and that can be easily captured using digital imaging equipment. Thus, leaves have become the most used morphological feature when performing automated plant classification and retrieval tasks using artificial intelligence [18]. Phenotypic diversity assessment and characterization using high-throughput phenotypes are considered more sensitive and cost-effective than traditional phenotypes [19,20].

The leaf morphology of grape (*V. vinifera* L.) is of multifaceted importance and is, therefore, the focus of this research. In addition to physiological and cultural relevance, taxonomic studies have largely depended on morphological leaf characteristics [13]. The morphological variation of grapevines can be explained not only by origin [21] and heredity [22] but also by climatic conditions and cultivation practices [23]. Given the high morphological variability of leaves between and within grape genotypes, analyses of phenotypes for predictive models have been a challenge. Accurately and consistently measuring grape leaf shape is essential for an in-depth phenotypic analysis of various growth and development parameters. Tomato Analyzer (TA) software 2.2 can measure close to 30 characteristics of two-dimensional shapes in a semi-automated way. Applying TA to grape germplasm resources is highly valuable for analyzing leaf phenotypes and collecting precise data. In addition, at present, the mining of candidate genes related to plant leaf shape is mainly based on mutants [24,25] and constructed hybrid populations [26], while genome-wide association study (GWAS) is less used. To explore more candidate genes related to grape leaf shape, we used the traits analyzed by TA software as target traits and conducted a GWAS in natural populations. Our research findings provide a theoretical reference for cultivating grape varieties with specific leaf shapes that can adapt to climate change.

## 2. Results

### 2.1. Changes in Grape Leaf Shape Character Parameters Determined Using TA

**Figure 1:**
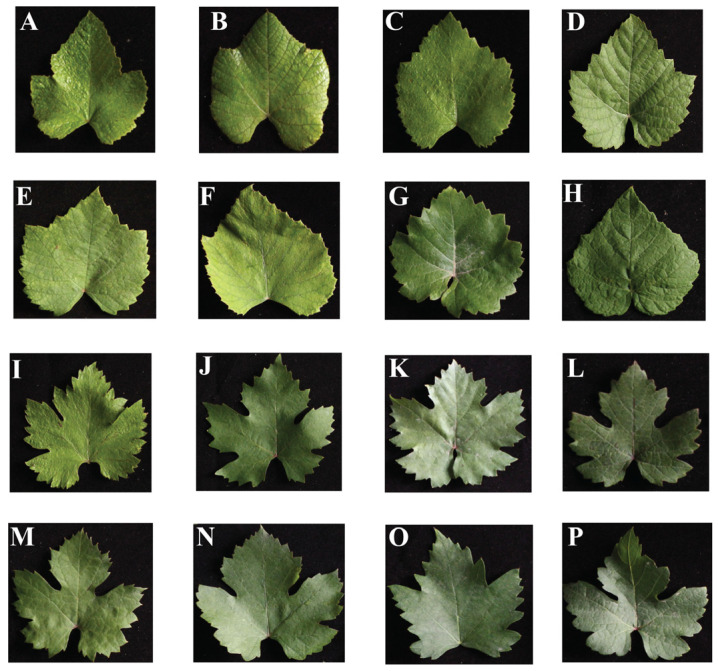
Leaf morphology of grape germplasm resources. (**A**) ‘Baixiangjiao’; (**B**) ‘Muscat Bailey’; (**C**) ‘Hartford’; (**D**) ‘Zhuosexiang’; (**E**) ‘Mudanhong’; (**F**) ‘Campbell’; (**G**) ‘Bulajinnie’; (**H**) ‘Canadice’; (**I**) ‘Zhuangyuanhong’; (**J**) ‘Fangxiang Grape’; (**K**) ‘Guibao’; (**L**) ‘Kutesaita’; (**M**) ‘Skendber’g; (**N**) ‘Jingyu’; (**O**) ‘Riluweijie’; (**P**) ‘Zaojinxiang’.

**Figure 2:**
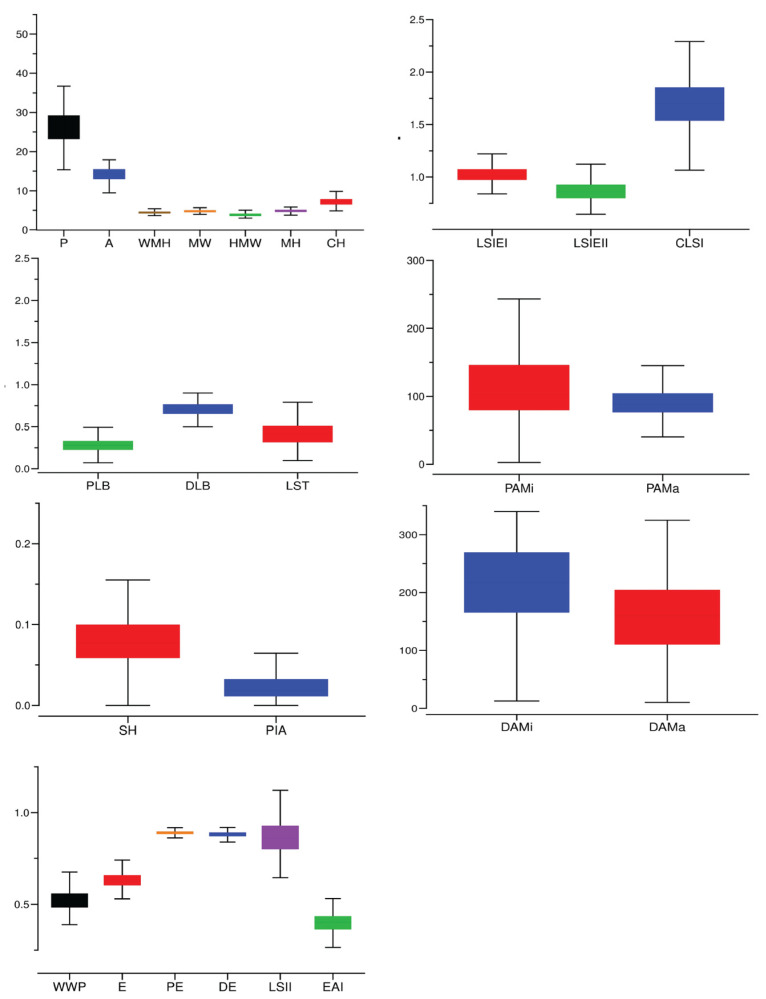
Different morphological grape leaf traits. A: area; CH: curved height; CLSI: curved leaf shape index; DAMa: distal-angle macro; DAMi: distal-angle micro; DE: distal eccentricity; DLB: distal leaf blockiness; E: eccentricity; EAI: eccentricity area index; HMW: height mid-width; LSIEI: leaf shape index external I; LSIEII: leaf shape index external II; LSII: leaf shape index internal; LST: leaf shape triangle; MH: maximum height; MW: maximum width; P: perimeter; PAMa: proximal-angle macro; PAMi: proximal-angle micro; PE: proximal eccentricity; PIA: proximal indentation area; PLB: proximal leaf blockiness; SH: shoulder height; WMH: width mid-height; WWP: width widest position. The whisker plot is used to summarize the distribution of the dataset. The boxes and vertical lines show minimum, maximum, and median values.

Differences in leaf morphology among grape germplasm resources are shown in Figure 1. Figure 2 presents a box diagram of the leaf shape trait-related parameters of these resources, highlighting the variations in these shape parameters. As shown in Table 1, the relevant parameters of leaf shape traits varied from 2.80% (shoulder height) to 118.37% (proximal-angle macro). Within these parameters, seven traits (shoulder height, width mid-height, proximal indentation area, eccentricity, distal eccentricity, distal-angle macro, and curved height) had a coefficient of variation less than 10%. Four traits (height mid-width, proximal eccentricity, maximum height, and leaf shape triangle) had a coefficient of variation greater than 40%, and the coefficients of variation for other traits ranged from 11.56% (distal-angle micro) to 36.32% (proximal-angle micro)

### 2.2. Principal Component Analysis and Correlation Analysis of Grape Leaf Shape Trait-Related Parameters

**Figure 3:**
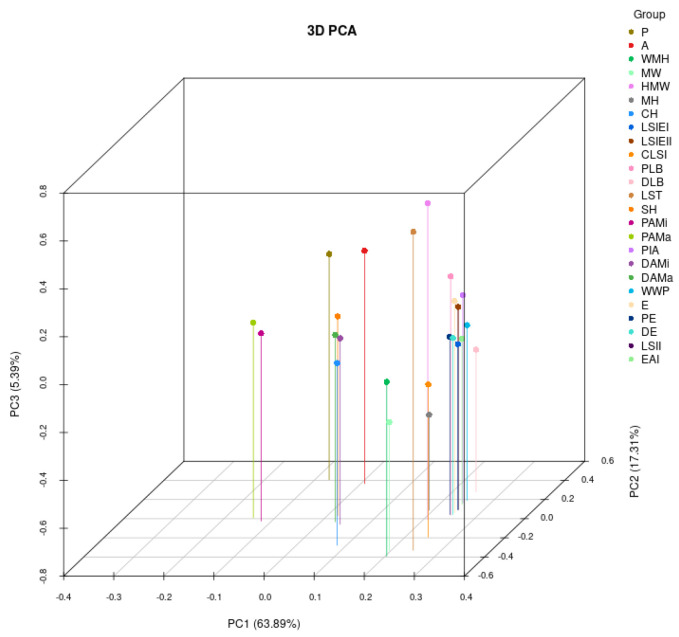
Principal component analysis (PCA) of grape leaf shape-related traits.

**Figure 4:**
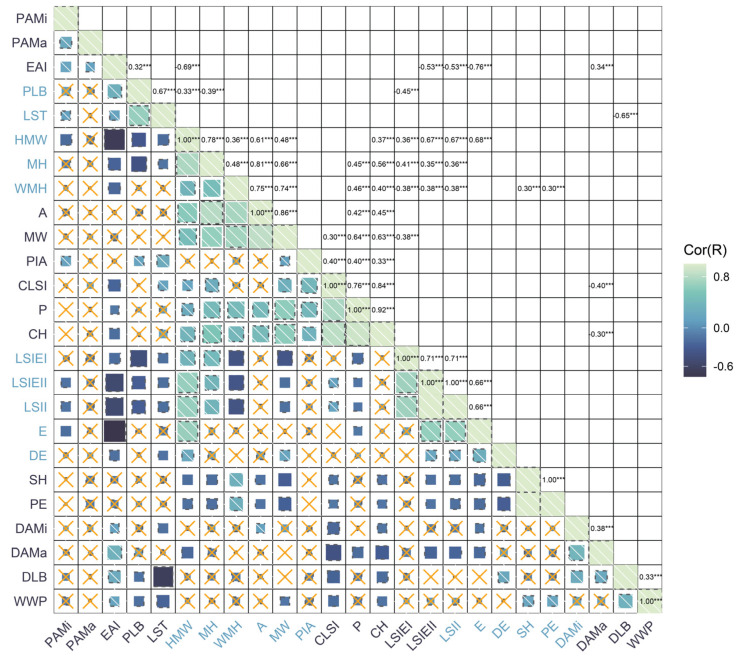
Correlation analysis of grape leaf shape-related parameters. The analyzed parameters include area (A), curved height (CH), and curved leaf shape index (CLSI), as well as both distal (DAMA) and proximal (PAMA) macro angles, and distal (DAMI) and proximal (PAMI) micro angles. Additional traits cover distal eccentricity (DE), proximal eccentricity (PE), eccentricity (E), and the eccentricity area index (EAI). Measurements such as height mid-width (HMW), maximum height (MH), maximum width (MW), and width mid-height (WMH) were also evaluated, alongside parameters like perimeter (P), proximal indentation area (PIA), proximal leaf blockiness (PLB), distal leaf blockiness (DLB), shoulder height (SH), and width at the widest position (WWP). Leaf shape indices include external I (LSIEI), external II (LSIEII), and internal (LSII), with the leaf shape triangle (LST) also accounted for in the analysis. The upper-right corner represents the correlation with a graph, *** represents the *p* value, and the lower-left corner represents the correlation with a number.

Figure 3 presents the Principal Component Analysis (PCA) of parameters related to grape leaf shape. The first three principal components each have eigenvalues exceeding 1, contributing to a cumulative variance of 86.59%. This high cumulative contribution rate suggests that these three components collectively account for almost 87% of the total variation observed in the dataset, indicating their significant explanatory power for the population. Therefore, the first three factors were extracted. The cumulative contribution rate for the previous principal component reached 63.89%. Analysis of correlations between parameters for the same leaf shape traits over two consecutive years (2023 and 2024) demonstrated a strong alignment in most traits. This high level of correlation indicates that these leaf shape characteristics are likely to be highly heritable, as shown by their stability across both years. In addition, Figure 4 shows the correlation analysis of parameters related to grape leaf shape traits. A positive correlation was found among seven leaf characteristics, including area, curved height, height mid-width, maximum height, maximum width, mid-height width, and perimeter. The correlation coefficients for these traits varied from 0.36 to 0.92.

### 2.3. GWAS for Grape Leaf Traits

**Figure 5:**
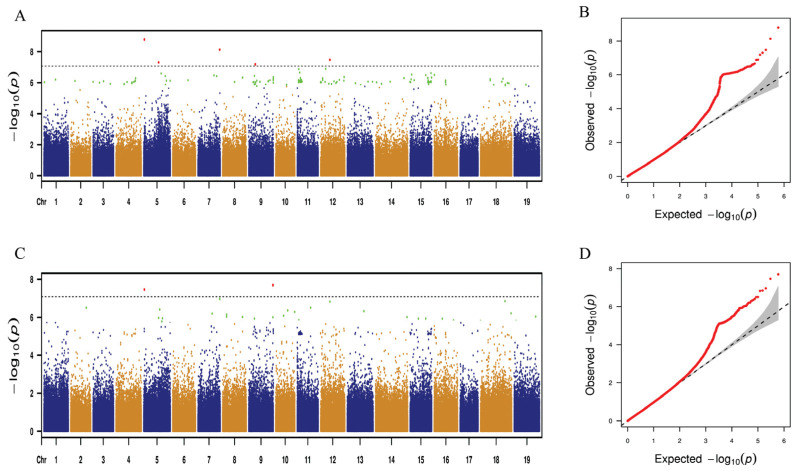
Genome-wide association study with the mixed linear model (MLM) for distal leaf blockiness. (**A**,**C**) Manhattan plots depicting SNP associations with distal leaf blockiness for 2023 and 2024, respectively. Thresholds are marked by the red and green dots, indicating significant cutoffs based on negative logarithms of 0.05 and 1 divided by total SNPs. SNPs with potential associations (candidate sites) lie above these threshold lines. (**B**,**D**) Quantile–quantile plots for distal leaf blockiness in 2023 and 2024, showing expected versus observed values. The abscissa indicates expected values, while the ordinate shows observed values.

**Figure 6:**
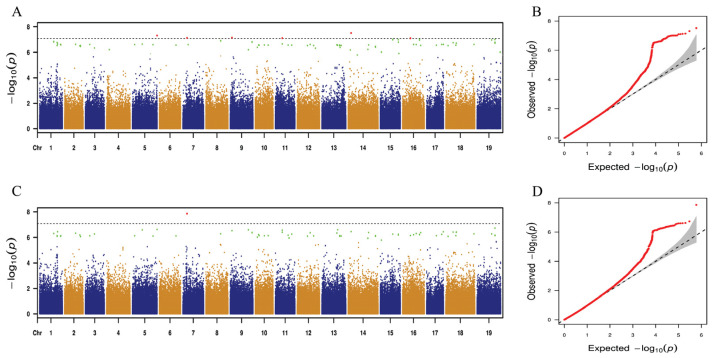
Genome-wide association study with the mixed linear model (MLM) for perimeter. (**A**,**C**) Manhattan plots depicting SNP associations with distal leaf blockiness for 2023 and 2024, respectively. Thresholds are marked by the red and green dots, indicating significant cutoffs based on negative logarithms of 0.05 and 1 divided by total SNPs. SNPs with potential associations (candidate sites) lie above these threshold lines. (**B**,**D**) Quantile–quantile plots for distal leaf blockiness in 2023 and 2024, showing expected versus observed values. The abscissa indicates expected values, while the ordinate shows observed values.

A mixed linear model (MLM) for GWAS was performed using 25 leaf traits. Using GWAS analysis, only two leaf traits, distinct leaf blockiness and perimeter, consistently mapped to the same SNP loci across both years. The remaining 23 traits, however, showed no such stable association with identical SNP loci over the two-year period. Figure 5 and Figure 6 show the GWAS results for distinct leaf blockiness and perimeter, respectively. The detailed results are presented in Tables S2 and S3. As shown in Figure 5 and Table S2, 163 and 36 SNP loci were significantly correlated with distinct leaf blockiness in 2023 and 2024, respectively. Further analysis revealed that for the 2 years, eight identical SNP loci were significantly correlated with distinct leaf blockiness, explaining 11.74–33.55% of phenotypic variation.

As shown in Figure 6 and Table S3, 91 and 83 SNP loci were significantly correlated with the perimeter trait in 2023 and 2024, respectively. Further analysis revealed a significant correlation between 81 identical SNP loci and perimeter for the 2 years, explaining 12.31–27.34% of phenotypic variation.

### 2.4. Distribution of SNP Loci Controlling Leaf Traits on Chromosomes

**Figure 7:**
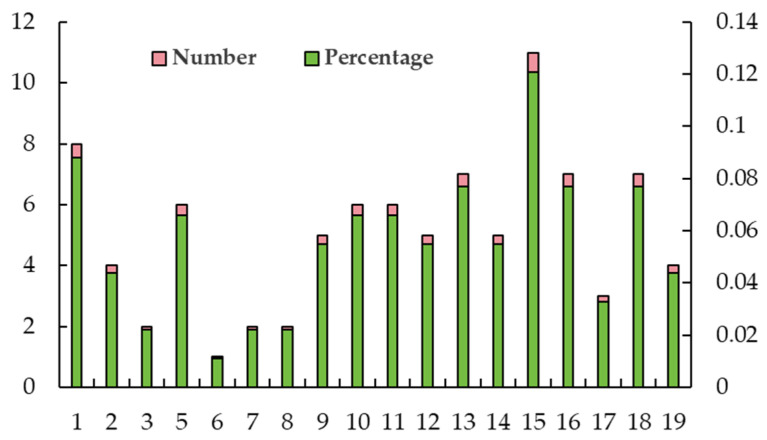
Distribution of SNP loci significantly associated with leaf traits on chromosomes over a 2-year period.

As shown in Figure 7, there was a significant correlation between 89 SNP loci and leaf traits during observations made in both years. These SNP loci were distributed on 18 chromosomes, except on chromosome 4. The highest number of SNP loci was distributed on chromosome 15. The distribution of SNP loci was lowest on chromosome 6, which contained just one SNP. In contrast, other chromosomes exhibited a range of SNP counts varying from two to eight.

### 2.5. Candidate Genes for Leaf Traits

The candidate genes for grape leaf shape traits were discovered in the present study (Table 2). These candidate genes include three cell-wall metabolism-related genes (VIT_01s0182g00160, VIT-05s0020g00420, and VIT_17s0053g00990), two plant hormone-related genes (VIT_15s0046g01050 and VIT_15s0048g00530), two genes related to ubiquitin ligase (VIT_03s0088g01090 and VIT_09s-0002g02020), two genes related to serine/threonine protein kinase (VIT_10s0003g01920 and VIT_10s0003g01920), one gene related to carbohydrate metabolism (VIT_14s0006g02720), two cell division-related genes (VIT_05s0029g-00040 and VIT_13s0047g00320), two transcription factor-related genes (VIT_11s0078g00480 and VIT_17s-0053g01010), and three other types of genes (VIT_05s0124g00250, VIT_14s0006g02400, and VIT_14s0006g02420). Among these 18 candidate genes for leaf traits, three genes (VIT_05s0029g00040, VIT_12s0178g00200, and VIT_15s0048g00530) are candidate genes for distinct leaf blockiness. The other 15 genes are candidate genes for the perimeter.

### 2.6. Enrichment Analysis of Candidate Genes for Grape Leaf Traits

**Figure 8:**
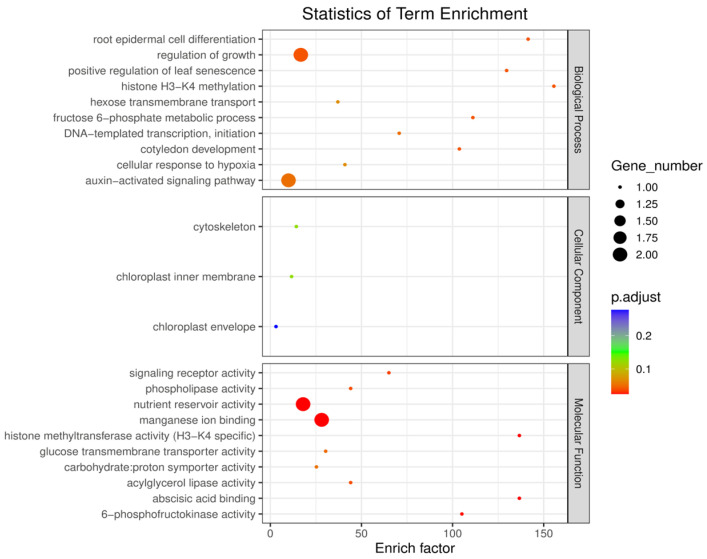
Analysis of GO terms associated with candidate genes for grape leaf traits. The size of each circle corresponds to the number of enriched genes in the pathway, with larger circles representing a higher count of genes. The colored circle indicates the *q* value, which reflects the adjusted *p* value.

We conducted Gene Ontology (GO) terminology and Kyoto Encyclopedia of Genes and Genomes (KEGG) enrichment analyses, as shown in Figure 8 and Figure S1, respectively, to understand the biological processes and pathways involved in the identification of candidate genes for grape leaf traits in this study. Enrichment analysis of the identified GO terms showed the potential roles of these candidate genes in leaf development. The biological process GO terms were mainly related to “regulation of growth”, “positive regulation of leaf senescence”, “histone H3-K4 methylation”, “hexose transmembrane transport”, “fructose 6-phosphate metabolic process”, “cotyledon development”, and the “auxin-activated signaling pathway”.

Cellular component GO terms were mainly related to “cytoskeleton”, “chloroplast inner membrane”, and “chloroplast envelope”. Molecular function GO terms were mainly related to “signaling receptor activity”, “phospholipase activity nutrient reservoir activity”, “manganese ion binding”, “histone methyltransferase activity (H3-K4 specific)”, “glucose transmembrane transporter activity”, “carbohydrate: proton symporter activity”, “acylglycerol lipase activity”, “abscisic acid binding”, and “6-phosphofructokinase activity”. As shown in Figure S1, KEGG pathway analysis of candidate genes for grape leaf traits showed that these candidate genes were mainly enriched in “RNA degradation”, “MAPK signaling pathway-plant”, “glycolysis/gluconeogenesis”, “plant hormone signal transduction”, “pentose phosphate pathway”, “galactose metabolism”, “fructose and mannose metabolism”, and “basal transcription factors”.

### 2.7. Tissue-Specific Expression Analysis of Candidate Genes

**Figure 9:**
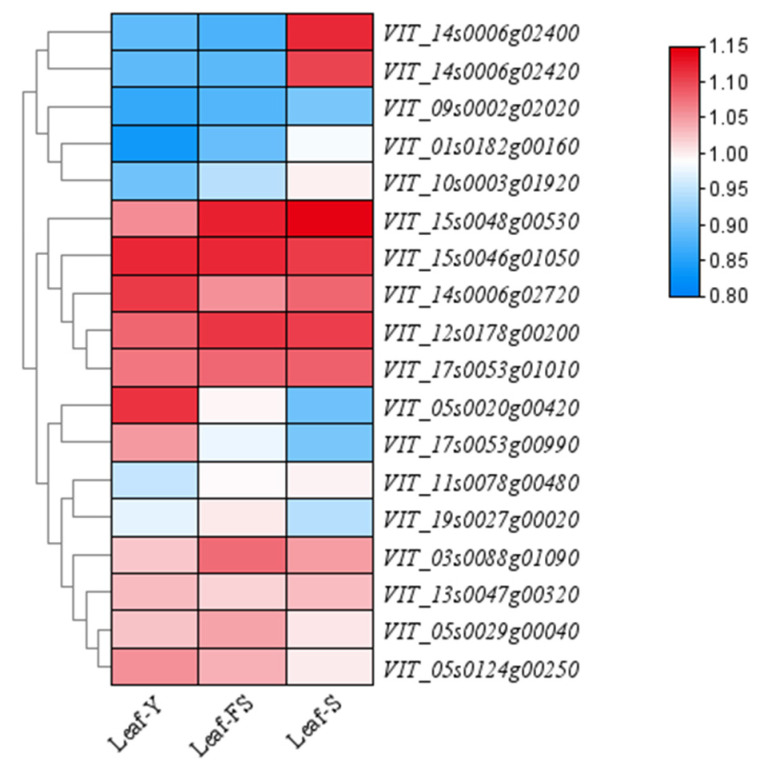
Expression dynamics of candidate genes associated with leaf shape traits. Leaf-Y: young leaf from shoots at the five-leaf stage; Leaf-FS: mature leaf from shoots at fruit set; Leaf-S: leaf senescence stage.

Candidate gene expression profiling was performed for grape leaf traits using the GEO dataset (No. GSE36128; [27]), as shown in Figure 9. These candidate genes related to leaf traits were expressed to varying degrees at different stages of leaf development, such as in young, mature, and senescent leaves. Therefore, these genes served as candidate genes for grape leaf traits.

### 2.8. Haplotype Analysis of Candidate Genes Related to Grape Leaf Traits

**Figure 10:**
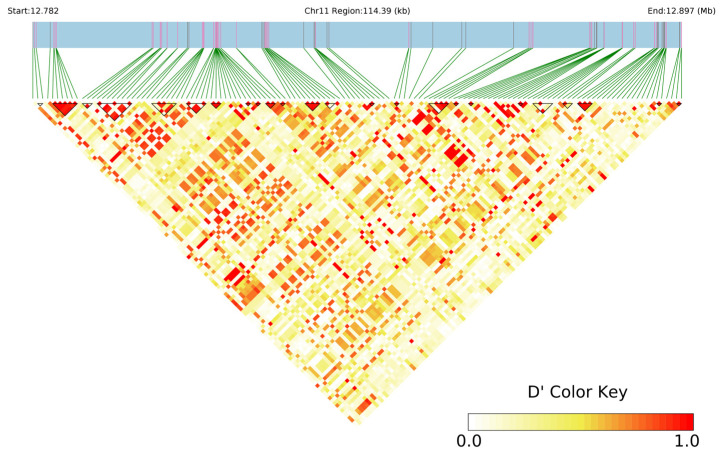
Haplotype analysis related to grape leaf traits (perimeter). Notes: LD regions are associated with Marke 17_122346 on chromosome 11. The yellow and red squares above are visualizations of LD values, with each square representing the LD results of two SNPs. The lighter the color, the smaller the LD value. If the LD between adjacent SNPs is greater than a certain threshold, then it forms a block.

Haplotype analysis was performed on some associated loci of grape leaf morphological traits (perimeter). Marker 17_122346 on chromosome 11 was detected within 2 years’ results and had high phenotypic variation; therefore, it was selected for haplotype analysis. Twenty-four haplotype blocks were obtained (Figure 10).

**Figure 11:**
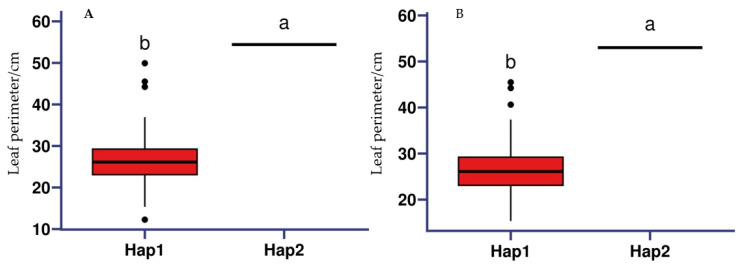
Boxplots for grape leaf perimeter based on haplotype (Hap). (**A**) 2023; (**B**) 2024. Different small letters in the figure represent significant differences (*p* < 0.05) between haplotypes of the same trait.

Haplotype analysis was performed on the promoter-region SNPs of the VIT_11s0078g00480 gene, which was jointly detected and found to control leaf perimeter-related traits over a period of 2 years. The haplotype analysis results showed that VIT_11s0078g00480 was divided into two haplotypes by the SNP in the promoter region. The VIT_11s0078g00480 gene showed significant differences in leaf perimeter among different haplotypes, with the leaf perimeter being significantly larger in Hap 2 than in Hap 1 (Figure 11).

## 3. Discussion

### 3.1. TA Greatly Expands Its Application Range in Measuring Plant Phenotype and Organ Morphology and Identifying Leaf Traits of Grape Germplasm Resources

Accurate and high-throughput assessment of plant organ morphology is challenging due to the quantitative nature of these traits and the often subjective methods used for their measurement [28]. TA software aims to identify objects of a specific size and image resolution, measured in dots per inch (pixels). For example, TA has been successfully applied to characterize the fruit morphological characteristics of tomatoes [29,30], eggplants [31], and peppers [32]. In genetic research, TA output has been used to detect quantitative trait loci for fruit shape in several isolated populations derived from hybridization between different cultivated tomato varieties and wild species [33,34,35]. In addition, the output generated by TA can be used to analyze other plant organ traits, such as the morphological characteristics of leaves and seeds [36,37]. The high expression of tomato fruit shape gene *SUN* leads to elongated fruits [38]. Using TA, the shape of leaves and cotyledons was measured in strains expressing *SUN* at high levels, demonstrating that this application can also be effectively used to measure the morphology of other plant organs. In summary, TA has become a key tool for objectively and reliably evaluating morphological variations in plant organs [36,37,38]. However, there have been no reports on the use of TA to analyze the morphology of grape leaves. In this study, we analyzed 25 grape leaf shape-related traits from 279 varieties using TA. We found that the coefficient of variation related to leaf traits ranged from 2.80% to 118.37%. Among the 25 leaf traits, the coefficient of variation for shoulder height and width mid-height was relatively small, while the coefficient of variation of the proportional-angle macro and leaf shape triangle largely varied. For the other leaf traits, the degree of variation ranged from 7.29% to 47.61%. With respect to the study of candidate genes for horticultural crop organs using TA-measured traits, more research on fruit traits in tomatoes is reported in [29,35]. However, few studies have detected candidate genes related to grape leaf shape based on TA analysis of traits.

### 3.2. GWAS of Grape Leaf Shape Trait-Related Genes

In this study, we used GWAS and TA to analyze grape leaf traits as 25 target traits and to identify candidate genes that control grape leaf traits. The relevant candidate genes discovered in this study included genes related to plant hormones, ubiquitin ligases, and transcription factors. Plant hormone auxin regulates many developmental processes in leaves. Leaf growth is promoted by plant hormones auxin [39], gibberellin (GA), and brassinolide (BR) [4]. In this study, we identified two plant hormone-related genes related to grape leaf shape traits, namely *VIT_15s0046g01050* (abscisic acid receptor PYL9) and *VIT_15s0048g00530* (auxin-responsive protein SAUR36), which were identified as candidate genes for the perimeter and distinct leaf blockiness traits, respectively. Auxins play a crucial role in leaf growth and development, regulating the initiation, formation, shape, and size of leaves [40,41]. Plant hormone auxin regulates many aspects of plant growth and development. Early auxin-responsive genes mediate their genomic effects on plant growth and development [42]. Most early-auxin responsive genes are divided into three families: *AUXIN/INDOLE ACETIC ACIDs* (*AUX/IAAs*), *GRETCHEN HAGEN3s* (*GH3s*), and *SMART AUXIN UP RNAs* (*SAURs*) [42,43]. *SAURs* were discovered in 1987 and are the largest family of early auxin-responsive genes [42]. Multiple studies suggest that *SAURs* regulate leaf growth by controlling cell expansion or division, which helps auxin regulate leaf growth and development [42]. In *Arabidopsis thaliana*, researchers found that *SAUR19* subfamily genes positively regulate leaf growth [44]. *SAUR19* and *EXPANSIN10* are the only genes that specifically affect the expansion of *A. thaliana* leaf cells rather than cell division [45]. However, how candidate gene *VIT_15s0048g00530* (auxin-responsive protein SAUR36), as mined in this study, regulates auxin content and, thus, affects grape leaf development requires further research.

Abscisic acid (ABA) was discovered half a century ago [46]. ABA also plays a critical role in plant growth and development, including during embryonic, seed, and seedling development [47] and seed dormancy [48,49]. In addition, it can promote leaf shedding [50]. ABA receptor PYL9 promotes drought resistance and leaf senescence in *A. thaliana* [51]. ABA receptors PYL9 and PYL8 play important roles in regulating lateral root growth in *A. thaliana* [52]. There has been no literature report on whether ABA receptor PYL9 can regulate plant leaf development. Although the ABA signaling pathway has been characterized in *Vitis vinifera* [53,54], there are few reports on its involvement in regulating grape plant growth and development, as well as its impact on biotic and abiotic stress. For example, studies have shown that overexpression of grape ABA receptor gene *VaPYL4* enhances *A. thaliana’s* tolerance to various abiotic stresses [55]. Candidate gene *VIT_15s0046g01050* (abscisic acid receptor PYL9) for the perimeter of grapes was identified in this study. However, the mechanism of leaf development regulation requires further study.

Ubiquitination is a refined post-translational modification that is widely present in all eukaryotes, including ubiquitin-activating enzymes (E1), ubiquitin-binding enzymes (E2), and ubiquitin-ligases (E3). Ubiquitin receptor DA1, E3 ubiquitin ligase DA2, and *ENHANCER OF DA1-1* (*EOD1*) (i.e., *BIG BROTHER* or *BB*) provide a source of control over leaf size and can limit the duration of cell proliferation [4]. The DA1-1 allele encodes a mutated DA1 protein (DA1^R358K^) that has a negative effect on *da1* and *da1*-related (DAR1), and plants that carry the *da1-1* mutation or knockout of both *DA1* and *DAR1* form leaves of varying sizes [56,57]. *DA1* encodes a ubiquitin-dependent protease that negatively regulates organ size [56,57]. *Arabidopsis da1-1* mutants produce large leaves, flowers, and seeds [57].

In this study, we identified two genes associated with ubiquitin ligase for grape leaf shape traits, namely *VIT_03s0088g01090* (RING finger protein 44) and *VIT_09s0002g02020* (putative F-box/LRR-repeat protein At5g02700). These genes were identified as candidates for the perimeter trait. A number of studies have confirmed the key role of RING-type E3s in different plant development processes, including seed germination, post-germination growth, and organ size determination [58]. In addition, there are more F-box genes in *Arabidopsis*, and studies have shown that some of these F-box genes are involved in plant hormone signaling pathways and plant developmental responses [58]. For example, F-box protein MAX2 (MORE AXILLARY GROWTH 2) is involved in the karrikin (KAR) and SL signaling pathways to regulate plant structure, photomorphogenesis, and leaf aging [59,60]. These results suggest that RING finger protein and F-box protein play important roles in the regulation of leaf development. Combined with our results, it can be reasonably speculated that *VIT_03s0088g01090* and *VIT_09s0002g02020* may play important roles in regulating grape blade perimeter size.

In this study, we identified two novel transcription factor-related genes associated with grape leaf shape traits: *VIT_11s0078g00480* (myb-related protein Myb4) and *VIT_17s0053g01010* (transcription initiation factor *IIB-2*). These genes were identified as candidates for the perimeter trait. Our research indicates that *VIT_11s0078g00480* (myb related protein Myb4) has two haplotypes and can be expressed normally in leaves, suggesting that this gene may be involved in the morphogenesis of grape leaves. Recent studies have shown that *CgMYB4* actively participates in regulating cell division and fiber differentiation during the early stages of stamen development in *Chelone glabra* L. [61]. Other *R2R3-MYB* genes have been shown to play important roles in tissue-specific differentiation of plants. For example, *GL1* in *A. thaliana* regulates leaf trichome differentiation [62], *AtMYB23* regulates cell-fate specificity in *A. thaliana* root epidermis [63,64], and *WEREWOLF* regulates *A. thaliana* epidermal cell patterns [64,65]. Based on the above studies, we speculate that *VIT_11s0078g00480* (myb-related protein Myb4) may also play an important role in regulating grape leaf morphology. Transcription factor IIB (TFIIB) is a general-purpose transcription factor of Pol II with only two cognates in most eukaryotes, namely Rrn7 and Brf of Pol I and Pol III, respectively [66]. In general, there are few reports on the regulation of plant organs by *Myb4* and *TFIIB2*. Further research is needed to understand how the candidate genes associated with perimeter (*VIT_11s0078g00480* and *VIT_17s0053g01010*) influence the regulation of grape leaf shape

In addition, in this study, we identified two genes associated with cell division in grape leaf shape traits, namely *VIT_05s0029g00040* (cyclin-dependent kinase inhibitor 5) and *VIT_13s0047g00320* (cell division cycle protein 123 homolog). We also identified two serine/threonine protein kinase-related genes associated with grape leaf shape traits, namely *VIT_10s0003g01920* (probable LRR receptor-like serine/threonine-protein kinase At1g07650 isoform) X1) and *VIT_10s0003g01920* (probable LRR receptor-like serine/threonine-protein kinase At1g07650 isoform X1). These four genes were identified as candidates for the perimeter trait. The in-depth molecular mechanism of these candidate genes in regulating grape leaf shape is also worthy of further study.

## 4. Materials and Methods

### 4.1. Grape Resources and Sample Collection

A total of 279 grape germplasm resources were selected for this study, and Table S1 shows the variety names. These varieties were stored in the experimental nursery of Xinjiang Academy of Agricultural Sciences (87°30′ E, 43°57′ N) in Anningqu, Urumqi, Xinjiang; the experimental nursery of Xinjiang Grape and Melon Research Institute (42°54′ E, 90°17′ N); and the grape Germplasm Resource Nursery of Zhengzhou Fruit Research Institute, Chinese Academy of Agricultural Sciences (113°39′ E, 34°43′ N). These varieties included 205 *V. vinifera* L. and 74 *V. vinifera* × *V. labrusca* varieties. Viticulture and management methods were described in our previous work [25]. The sampling method for grape leaves followed the method described in a previous report [13]. Most varieties were represented by two cloned grapevines, with two to three leaves sampled from each vine. Five leaves were collected from most of the grape varieties included in the current research. If possible, one sample was collected from the midpoint of two branches on each vine, and the leaves were continuously collected from similar developmental stages. Leaf sampling was performed from 8:00 AM to 10:00 AM. In both 2023 and 2024, leaf blades that exhibited similar maturity and were free of surface defects were chosen for testing.

### 4.2. Relevant Parameters of Grape Leaf Shape Were Analyzed Using TA

We randomly selected five leaves and captured photographs of the samples with reference to the formula described in a previous report [29]. We used TA 3.0 to measure 25 leaf morphology indicators. These metrics included seven basic measurement-related metrics (area, perimeter, curved height, maximum width, width mid-height, height mid-width, and maximum height), one asymmetry-related metrics(width widest position), three blockiness-related metrics (distal leaf blockiness, leaf shape triangle, and proximal leaf blockiness), two metrics related to distal leaf end shape (distal-angle macro and distal-angle micro), three metrics related to the leaf shape index (curved leaf shape index, leaf shape index external I, and leaf shape index external II), five metrics related to internal eccentricity (distal eccentricity, eccentricity, eccentricity area index, leaf shape index internal, and proximal eccentricity), and four proximal leaf end shape-related metrics (proximal micro and macro angles, proximal indentation area, and shoulder height) [30,31].

### 4.3. Genome-Wide Association Study

The *Vitis vinifera* PN40024 genome was used as the reference genome [32]. Based on previous work, which included identifying single-nucleotide polymorphism (SNP) markers, analyzing the population structure and population-level linkage disequilibrium (LD) [28], we conducted a GWAS of 25 leaf traits analyzed using TA in the past 2 years (2023 and 2024). Referring to our previous work [28], ADMIXTURE software was used for population structure analysis.

PopLDdecay software (PopLDdecay-3.43) was used to analyze linkage disequilibrium (LD) at the population level, with parameters set as -MAF 0.05, -MaxDist 500, and -Miss 0.25 [33]. GWAS was performed based on SNPs using TASSEL software(TASSEL 5.2.94) [35] to obtain relevant values by compressing mixed linear models (MLMs). Among them, the sample population structure (Q) was calculated using hybrid software, while the relationship between K samples was determined using SPAGeDi software(1.0) [34,36]. MLMs utilized Q + K information. Finally, each SNP site was assigned a correlation value (*p*) [28].

### 4.4. Annotation and Function Prediction of Candidate Genes

According to the physical location of SNP markers significantly associated with leaf-related traits in the grape reference genome, candidate genes within the upper (UD) and lower (LD) decay distance regions of significant SNP sites were screened. Gene annotation and functional prediction were performed using the Clusters of Orthologous Groups (COG), Kyoto Encyclopedia of Genes and Genomes (KEGG), Gene Ontology (GO), Swiss-Prot, and non-redundant (NR) databases [28]. R software(Rstudio - 2023.06.1) with the clusterProfiler package was used to perform GO and KEGG pathway enrichment analyses of annotated grape leaf trait-related candidate genes.

### 4.5. Haplotype Analysis and Expression Analysis

Haplotype analysis was performed on the significantly associated loci identified using LDBlockShow (version 1.40). The expression levels of potential genes related to leaf characteristics at various developmental stages were analyzed using data from the comprehensive gene expression database (GEO dataset, No. GSE36128) [37]. The logarithm of the original value, based on 10, was used to generate a heatmap using TBtools software (TBtools-II) [38].

### 4.6. Statistical Analysis

Since most leaf shape traits were highly correlated over a 2-year period, we used SPSS 20.0 (IBM, Armonk, NY, USA) to analyze the variation of the mean values from the 2-year data. Pearson correlation coefficients were calculated using R with the ‘psych’ package and the ‘corr.test’ function and plotted using the ‘corrplot’ package. Principal component analysis (PCA) was performed in R using the ‘princomp’ function from the ‘stats’ package, with results summarized using the ‘summary’ function. For visual representation, box plots were generated using the ‘boxplot’ function in R

## 5. Conclusions

This study analyzed leaf shape trait-related genes using GWAS for the first time using 25 leaf trait parameters analyzed using TA, a high-throughput analysis software, to explore new key genes closely linked to grape leaf shape traits. The leaf trait-specific genes mined in this research included genes related to various molecular, cellular, and biological processes, including serine/threonine protein kinase, ubiquitin ligase, plant hormones, and carbohydrate metabolism. This research enhances our comprehension of the genetic regulation of leaf characteristics in grapes. Identifying candidate genes that are strongly associated with these leaf traits holds significant importance for the development of grape varieties with desired leaf shapes.

## Figures and Tables

**Table 1:** Variations in different leaf traits.

Characteristic	Maximum	Minimum	Mean	Standard Deviation	Coefficient of Variation
Area	53.71	14.10	26.49	4.86	18.36%
Curved leaf shape index	17.91	5.26	14.25	1.88	13.19%
Curved height	6.30	2.61	4.44	0.40	8.90%
Distal-angle macro	5.87	2.71	4.75	0.42	8.75%
Distal-angle micro	5.03	2.49	3.83	0.44	11.56%
Distal eccentricity	5.84	3.01	4.85	0.42	8.71%
Distal leaf blockiness	12.46	3.92	7.23	1.16	16.03%
Eccentricity	1.47	0.84	1.03	0.08	7.71%
Eccentricity area index	1.34	0.60	0.87	0.10	11.68%
Leaf shape index external I	2.61	1.07	1.71	0.26	15.06%
Leaf shape index external II	0.57	0.07	0.29	0.09	30.36%
Leaf shape index internal	0.95	0.16	0.70	0.10	14.49%
Leaf shape triangle	2.44	0.10	0.45	0.23	50.23%
Height mid-width	0.40	0.00	0.08	0.03	40.32%
Maximum height	316.20	2.63	116.41	55.43	47.61%
Maximum width	230.39	0.95	90.81	26.81	29.53%
Proximal-angle macro	0.27	0.00	0.03	0.03	118.37%
Proximal-angle micro	339.76	6.03	208.37	75.68	36.32%
Proximal eccentricity	324.73	10.37	159.97	68.56	42.85%
Proximal leaf blockiness	0.73	0.34	0.52	0.07	12.81%
Proximal indentation area	0.77	0.45	0.63	0.05	7.29%
Shoulder height	1.16	0.82	0.89	0.02	2.80%
Width mid-height	1.03	0.73	0.88	0.04	4.07%
Width widest position	1.34	0.57	0.87	0.10	11.74%
Perimeter	0.55	0.18	0.40	0.06	14.47%

**Table 2:** Candidate genes related to grape leaf traits.

Gene ID	Location	Nr Annotation
*VIT_01s0182g00160*	1:13463652–13467087	PREDICTED: galactoside 2-alpha-L-fucosyltransferase [*Vitis vinifera*]
*VIT_03s0088g01090*	3:9340147–9341749	PREDICTED: RING finger protein 44 [*Vitis vinifera*]
*VIT_05s0020g00420*	5:2340655–2343157	PREDICTED: polygalacturonase At1g48100 [*Vitis vinifera*]
*VIT_05s0029g00040*	5:14450243–14453228	PREDICTED: cyclin-dependent kinase inhibitor 5 [*Vitis vinifera*]
*VIT_05s0124g00250*	5:21154095–21220251	PREDICTED: histone-lysine N-methyltransferase ATX1 [*Vitis vinifera*]
*VIT_09s0002g02020*	9:1789739–1791953	PREDICTED: putative F-box/LRR-repeat protein At5g02700 [*Vitis vinifera*]
*VIT_10s0003g01920*	10:6997420–7015431	PREDICTED: probable LRR receptor-like serine/threonine-protein kinase At1g07650 isoform X1 [*Vitis vinifera*]
*VIT_11s0078g00480*	11:15480609–15481984	PREDICTED: myb-related protein Myb4 [*Vitis vinifera*]
*VIT_12s0178g00200*	12:11429553–11433532	PREDICTED: actin-101 isoform X1 [*Vitis vinifera*]
*VIT_13s0047g00320*	13:16190297–16192565	PREDICTED: cell division cycle protein 123 homolog [*Vitis vinifera*]
*VIT_14s0006g02400*	14:19942477–19949902	PREDICTED: putative germin-like protein 2-1 [*Vitis vinifera*]
*VIT_14s0006g02420*	14:19962832–19963625	PREDICTED: putative germin-like protein 2-1 [*Vitis vinifera*]
*VIT_14s0006g02720*	14:20731846–20739925	PREDICTED: plastid hexose transporter isoform X1 [*Vitis vinifera*]
*VIT_15s0048g00530*	15:14642820–14643902	PREDICTED: auxin-responsive protein SAUR36 [*Vitis vinifera*]
*VIT_15s0046g01050*	15:18133272–18136212	PREDICTED: abscisic acid receptor PYL9 [*Vitis vinifera*]
*VIT_17s0053g00990*	17:17733901–17735829	PREDICTED: expansin-A10 [*Vitis vinifera*]
*VIT_17s0053g01010*	17:17937970–17944266	PREDICTED: transcription initiation factor IIB-2 [*Vitis vinifera*]
*VIT_19s0027g00020*	19:18803078–18804735	PREDICTED: serine/threonine-protein kinase WAG1 [*Vitis vinifera*]

## Data Availability

Detailed data is provided in supplementary files.

## Supplementary Materials

The following supporting information can be downloaded at: https://www.mdpi.com/article/10.3390/ijms252212101/s1.
