# Supplementary material for: Mining of Candidate Genes Associated with Leaf Shape Traits in Grapes
Source: Int J Mol Sci. 2024 Nov 11;25(22):12101. doi: 10.3390/ijms252212101 (PMC11593594; doi:10.3390/ijms252212101)
Supplement: Supplementary file 1 [file ijms-25-12101-s001.zip › Figure S1.pdf]

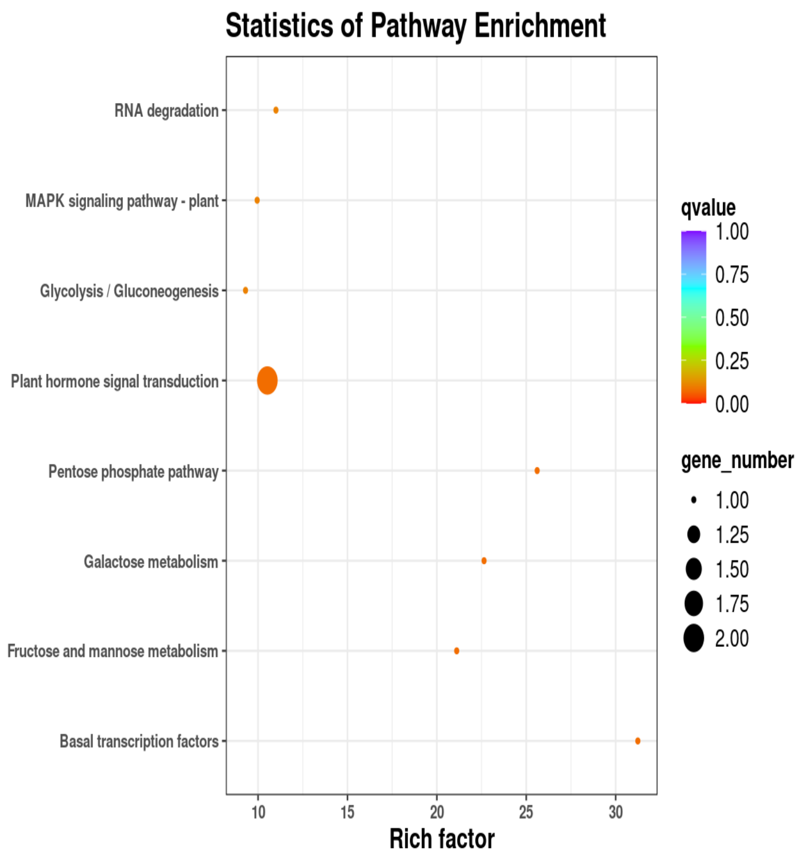

Fig. S1. Analysis of KEGG enrichment analysis associated with candidate genes for grape leaf traits
