# Supplementary material for: Mining of Candidate Genes Associated with Leaf Shape Traits in Grapes
Source: Int J Mol Sci. 2024 Nov 11;25(22):12101. doi: 10.3390/ijms252212101 (PMC11593594; doi:10.3390/ijms252212101)
Supplement: Supplementary file 1 [file ijms-25-12101-s001.zip › Table S1.pdf]

Table S1. Experimental material

Species	Varieties
V. vinifera × *V. labrusca*	Cuifeng, Aierweiyin, Bath, Baixiangjiao, Muscat Bailey, Lady Washington, Cuihong, Daban 48202, Fenghou, Fengshou, Takasumi, Takasago, Hartford, Black Olympia, Herbert, Honey Black, Red Olympia, Beni Fuji, Beni Zuiho, Suffolk Red, Hongshaungwei, Hongxiangjiao, Hongxing, Beniizu, Hupo, Hutai 8, Jifeng, Golden Muscat, Jingya, Jingyou, Kyoho, Baiaolin, Jumeigui, Catawba, Campbell, Zhuangyuanhong, Ryuho, New York Mascat, Rommel, Meizhoubai, Mills, Honey Red, Mizhi, Moldova, Niagara, Sagami, Shenxiu, Steuben, Fujiminori, Muscat Angel, Tano Black, Tano Red, Vergennes, Xiyanghong, Xianfeng, Xiangyue, Xinnonghong, Izu Nishiki, Zhuosexiang, Shigyoku, Zizao, Zizhenxiang, Hupei 2,Urbana, Russian Concord, Canada Muscat, Otoda, Geerbi, Governor Rose, Shennong Shuofeng, Shennong Xiangfeng, Rosebell, Heijianiang
V. vinifera L.	Guifei Rose, Honglianzi, Rose Cioutat, Crimson Seedless, Jade Seedless, 87-1, 90-1, 11-43, Angour siokh, Madeleine Angevine, Annamaria, Augusta, Irsay Oliver, Olympia, Baidalayi, Khalili Blanc, Khoussaine Blanc, Baijixin, White Rozaki, Afuali, Boulgal, Skendberg, Rosario Bianco, Julski Beaser, Bulajinnie, Bulieqiluoranxi, Chaobao, Djoura ousioum, Misket Dounvaski, Fangxiang, Mathias Aromatic, Fangxiang Grape, Cardinal, Fenhong Alimandeng, Fenhong Lachaji, Fenhong Yayisuna, Fenghuang 51, High Bailey, Greaca, Guibao, Hatebaer, Hetianhong, Black Hamburg, Heijixin, Heimo Pensal Blanco, Heixiani, Gousale Kara, Hungaria, Rosario Rosso, Flame Muscat, Ruby Okuyama, Huangmisi, Jilaer, Jinan Zaohong, Jingkejing, Kelimukaonisong, Xycahhe Kejihm Bapmak, Kutesaita, Lizixiang, Rizamat, Longyan, Lünai, Gros Colman, Rodi, Manai, Precoce de Malingre, Manao, Mascat Hamburg, Manicure Finger, Mihaer, Mudanhong, Jingyu, Naduoer, Niuxin, Paikaer, Aromatic of Pecs, Pannuoniya, Pinger, Queen of Vineyard, Mascat Plevenski, Qichakapulie, Qiaqiwahe, Qiaobao 2, Qiaowushi, Joanna Charnice, Qiubai, Riluweijie, Centenial, Shenyang Rose, Shengli, Shenglihuayao 2, Christmas Rose, Su 46, Sultanick Rose, Madeleine Solomon, Tebieheidali, Weihongbai, Victoria, Wujiakeayi, Selecta, Xiabai, Xiangfei, Xiaobai, Muscat Mathiasz Janosne, Yang Grape, Kocsias Irma, Yilixiang, Elisabeth Grape, Ispissar, Italia, Rose D Italia, Zaoheibao, Zaojinxiang, Zaomanao, Zaomeigui, Zexiang, Zeyu, Zhengzhou Zaohong, Zifeng, Zijixin, Zitao, Zizhenzhu, Emberad, Aishen Rose, Otilia, Queenora, Perlette, Madh Pa Fare, Bronx Seedless,Vanerssa Secdless, Flame Seedless, Ruby Seedless, Blush Seedless, Hongwuzilu, Mars, Jingfeng Seedless, Jingzaojing, Jingzijing, Kangnainuo, Canadice, Bixiang Seedless, Lakemont, Dawn Seedless, Beauty Seedless, Melissa Seedless, Nasaili, Autumn Seedless, Sangduohani, Thompsons Seedless, Rose Seedless, Honey Seedless, Black Monukka, Xiying, Ximulaode,

	Xinpu 2, Yanggeer, Guifuren, Qingzi, Tiankang Meigui, Waerse, Jufeng Meigui, Huafu, Lival, Xiwazi, Hongsidi, SG, Anmamaliya, Heifeng, Zuirenxiang, Sipahanbao, Golden Finger, Black Muscat, Scarlet Royal, Sweet Scarlet, Zaokangbao, Qiuhongbao, Waltham Cross, Qiuheibao, Sivan, Xiabai, Jintian Feicui, Huangjia Seedless, Jintianhong, Bujisuli, Zidiqiu, Star Light, Gold Star, Kamea, Mutant of Thompson Seedless, Shennong Jinhuanghou, Lilit, Gold, Pinot Blanc, Ugni Blanc, Cabernet Sauvignon, Blue French, Pinot Noir, Italian Riesling, Pinot Gris, Carignan, Riesling, Merlot, Cabernet Gernischet, Chardonnay, Muscat Blanc
